# Protocol for integrated phosphoproteomic and proteomic analysis of the mouse ventral tegmental area with functional and kinase motif enrichment

**DOI:** 10.1016/j.xpro.2026.104911

**Published:** 2026-10-20

**Authors:** Cody C. Diezel, Paul R. Langlais, Todd W. Vanderah, Arthur C. Riegel

**Affiliations:** 1 Comprehensive Center for Pain and Addiction (CCPA), University of Arizona, Tucson, AZ 85724, USA; 2 Department of Pharmacology, College of Medicine, University of Arizona, Tucson, AZ 85724, USA; 3 Department of Medicine, Division of Endocrinology, University of Arizona, Tucson, AZ 85724, USA; 4 Department of Neuroscience, College of Science, University of Arizona, Tucson, AZ 85724, USA; 5 James C. Wyant College of Optical Sciences, University of Arizona, Tucson, AZ 85724, USA

**Keywords:** Bioinformatics, Molecular Biology, Neuroscience, Proteomics

## Abstract

We present a protocol for label-free proteomic and phosphoproteomic analysis of the mouse ventral tegmental area (VTA). We describe steps for combining region-specific biopsy-punch collection, parallel proteomic and phosphoproteomic processing, liquid chromatography-tandem mass spectrometry (LC-MS/MS), and downstream enrichment analysis. We detail procedures for SynGO-based synaptic localization, g:Profiler/DAVID pathway enrichment, and iceLogo/pLogo motif analysis. This protocol enables quantitative assessment of protein abundance, phosphorylation dynamics, and functional interpretation from limited brain region samples.

For complete details on the use and execution of this protocol, please refer to Diezel et al.^1^

## Highlights

• Steps for collecting and homogenizing mouse VTA tissue
• Instructions for in-gel digestion and SMOAC phosphopeptide enrichment
• Steps for LC-MS/MS analysis of proteome and phosphoproteome samples
• Guidance on functional enrichment and kinase motif inference

## Abstract (editor highlights)

Publisher’s note: Undertaking any experimental protocol requires adherence to local institutional guidelines for laboratory safety and ethics.

## Graphical abstract

**Figure:**
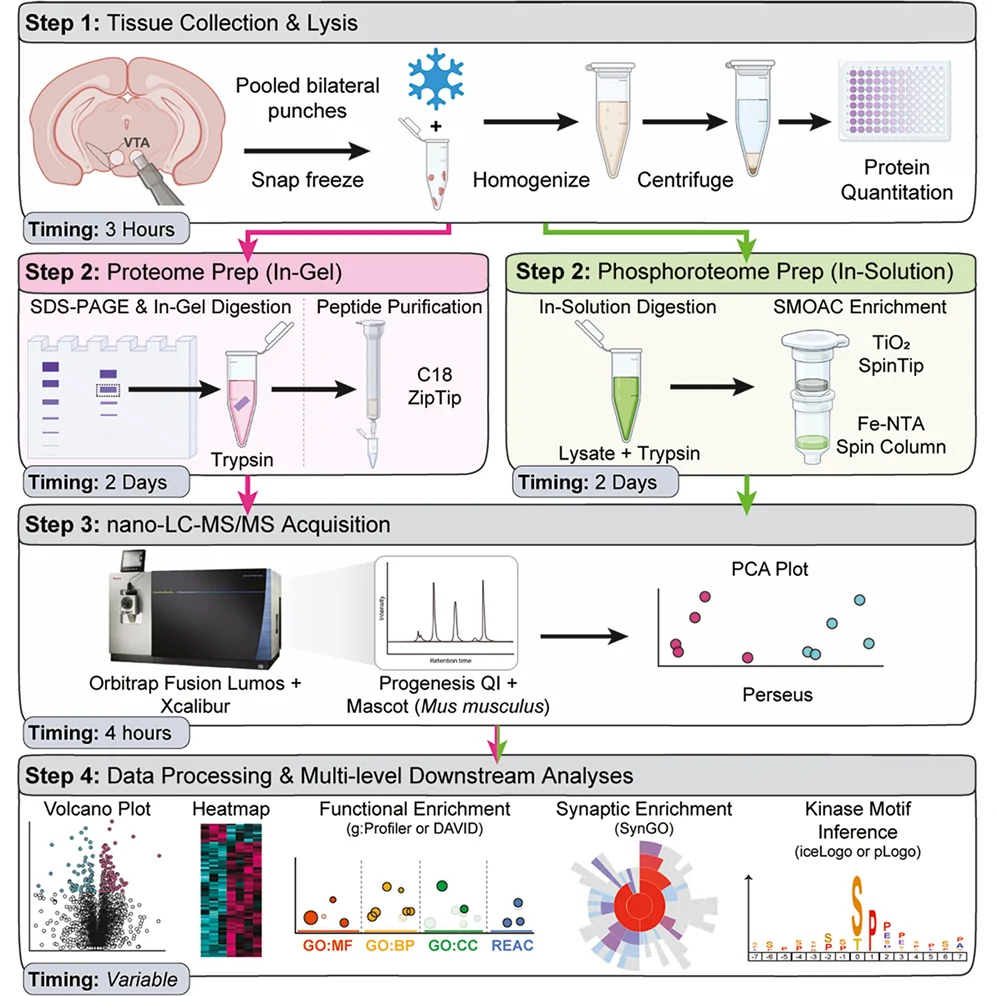

## Before you begin

This protocol details the collection, processing, mass spectrometry analysis, and downstream functional enrichment of proteins and phosphopeptides from the mouse ventral tegmental area (VTA). It covers biopsy punch collection of the VTA, digitonin-supplemented RIPA protein extraction, and two independent processing workflows: in-gel digestion for proteome-wide analysis and in-solution digestion with metal oxide affinity chromatography (SMOAC) for phosphopeptide enrichment, each followed by nano-LC-MS/MS on an Orbitrap Fusion Lumos system and label-free quantification. Rather than stopping at lists of changing molecules, this workflow moves from protein identification to functional insight by first establishing which proteins are altered between experimental groups, then revealing how those proteins are regulated through phosphorylation state changes to show which signaling pathways experience changes in activity through modification rather than through changes in total abundance alone. From there, functional enrichment analyses place these protein and phosphoprotein changes into their broader biological context by determining which cellular compartments and biological processes are affected. Identification of enriched phosphopeptide sequence motif helps infer candidate upstream kinase families and generate hypotheses for follow-up validation. Together, this integrated approach transforms lists of changing molecules into testable mechanistic hypotheses about the biological processes altered in experimental models.

The steps below reflect the workflow used in our associated study,^1^ which profiled VTA proteomic and phosphoproteomic remodeling at 1 week and 4 weeks after partial sciatic nerve ligation (pSNL), and at 30 min after intracerebroventricular (ICV) infusion of a CaMK1 inhibitor, CS640. Bilateral VTA punches from C57BL/6J mice were pooled (2 punches per sample for proteomics, 3 for phosphoproteomics) and processed through the workflows described herein to quantify label-free protein abundance, profile phosphopeptide dynamics, and perform downstream Gene Ontology (GO), Reactome, SynGO, and kinase-motif enrichment analyses. The associated study^1^ incorporated additional molecular analyses, including a targeted lipidomic assessment of VTA tissue using distinct sample-processing, mass-spectrometry, and analytical workflows. These analyses are therefore not included in the protocol described here.

The workflow is transferable to other mouse brain regions accessible by biopsy punch (e.g., nucleus accumbens, dorsal striatum, hippocampus, prefrontal cortex) and to other rodent models of neurological or psychiatric diseases in which broader functional changes are of interest. The in-solution digestion and post translational modification (PTM) enrichment strategy should be adaptable to other PTMs (acetylation,^2^^,^^3^ ubiquitination,^4^ glycosylation,^5^ etc.) by substituting the enrichment step with the appropriate PTM-specific enrichment methods along with upstream lysis, digestion, and LC-MS/MS parameter optimization; however, these applications and optimization steps were not evaluated in the present protocol. Alternative downstream tools (e.g., PhosR, ssGSEA, ESPD 2.0) may be substituted depending on the specific analysis goal and experimenter expertise.

### Innovation

Many phosphoproteomic and proteomic workflows emphasize differential abundance, but functional annotation is often needed to interpret coordinated biological changes, and such workflows are typically built for laboratories with dedicated proteomics expertise. This protocol details an integrated workflow for comprehensive proteome and phosphoproteome profiling from small mouse brain regions for neuroscience laboratories without that infrastructure. It addresses the challenge of limited tissue availability and proteomic analysis by presenting existing workflows in a cohesive pipeline with three linked features. First, the protocol embeds GO/Reactome enrichment, kinase-motif inference, and other major downstream analyses as standard workflow steps rather than optional post-processing since lists of changing molecules without biological context can be difficult to interpret and miss larger patterns of coordinated signaling changes. Second, the protocol applies a consistent sampling strategy based on 1 mm VTA biopsy punches. Bilateral samples are pooled to ensure adequate protein input for each proteomic analysis, with in-gel digestion providing total protein quantification and in-solution digestion combined with SMOAC enrichment to quantify phosphopeptides. Third, the protocol integrates the entire workflow from tissue collection through biological interpretation, with each step designed to preserve compatibility with subsequent processing and analysis. The quality of downstream interpretation depends on upstream processing quality— pooled bilateral punches ensure adequate protein input, digitonin-supplemented RIPA preserves membrane-associated synaptic proteins, and phosphatase inhibition preserves labile phosphorylation states. This produces interpretable biological hypotheses rather than lists of changing molecules, without requiring a specialized proteomics background.

### Institutional permissions

This protocol uses tissue collected from vertebrate animals. Researchers must obtain approval from their Institutional Animal Care and Use Committee. All experimental procedures were approved by the University of Arizona Animal Care Use Committee (Protocols 19–556, 19–600, and 2021–0804), in strict compliance with NIH guidelines for laboratory animal care.

### Preparation of lysis buffer

**Timing: 30 min**

1. Prepare the radioimmunoprecipitation assay (RIPA) base buffer containing 20 mM Tris-HCl, 150 mM NaCl, 2 mM EDTA, and 0.1% SDS.
  a. Mix all components in sterile distilled water.
  b. Adjust pH to 7.4 if necessary.
2. Supplement the base buffer with detergents and inhibitors immediately before use.
  a. Add Triton X-100, sodium deoxycholate (SDC), sodium orthovanadate (NaV), phenylmethylsulfonyl fluoride (PMSF), sodium fluoride (NaF), protease inhibitor cocktail, phosphatase inhibitor cocktail, and digitonin.
  b. Keep the supplemented buffer on ice and use it immediately.

**CRITICAL:** Add protease and phosphatase inhibitors (NaV, PMSF, NaF, protease inhibitor cocktail, phosphatase inhibitor cocktail) and digitonin immediately before use. Inactive inhibitors will result in protein degradation and dephosphorylation. PMSF has a half-life ranging from 30 to 110 min at 20–25°C in solution and will lose efficacy if use is delayed.^6^

### Preparation of dissection tools and animal scheduling

**Timing: 10 min**

3. Schedule animal sacrifices based on experimental timeline.
  a. Ensure a sufficient number of animals are available to pool.

***Note:*** The associated study pooled 2 bilateral punches per sample (total of 4 VTA punches) for proteomics or 3 bilateral punches per sample (total of 6 VTA punches) for phosphoproteomics.

4. Chill the brain matrix and collection tools (biopsy punch, razor blades) on ice or in a −20°C freezer prior to brain extraction.

***Note:*** Chill the matrix for at least 15 min before use.

**WARNING:** Dry ice chills the matrix rapidly, but tissue may stick if matrix is too cold.

5. Prepare the tissue collection area.
  a. Fill a container with ice and cover with foil.
  b. Gently push foil down so it is flat on the ice.

***Note:*** Other tools to assist the collection process (e.g., pre-chilled cold blocks, ice packs, etc.) are recommended where helpful.

6. Prepare containers for snap-freezing collected tissue samples.
  a. Fill a container with dry ice or liquid nitrogen.
  b. Label tubes for each sample group.

## Key resources table

**Table:** 

REAGENT or RESOURCE	SOURCE	IDENTIFIER
**Chemicals, peptides, and recombinant proteins**		

Digitonin	Sigma Aldrich (Burlington, MA, USA)	Cat.#: D141
Protease Inhibitor Cocktail	Sigma Aldrich (Burlington, MA, USA)	Cat.#: P8340
100X Phosphatase inhibitor	Selleckchem (Houston, TX, USA)	Cat.#: B15001
Pierce™ Lys-C Endoproteinase	ThermoFisher (Waltham, MA, USA)	Cat.#: 90307
Pierce™ Trypsin Protease	ThermoFisher (Waltham, MA, USA)	Cat.#: 90057
Pierce™ HeLa Protein Digest Standard	ThermoFisher (Waltham, MA, USA)	Cat.#: 88329
Pierce™ DTT (Dithiothreitol)	ThermoFisher (Waltham, MA, USA)	Cat.#: 20290
0.1% FA in Water	Fisher Chemical (Pittsburgh, PA, USA)	Cat.#: LS118-1
0.1% FA in Acetonitrile (ACN)	Fisher Chemical (Pittsburgh, PA, USA)	Cat.#: LS120-1

**Critical commercial assays**		

Pierce™ BCA Protein Assay Kit	ThermoFisher (Waltham, MA, USA)	Cat.#: 23225
High Select Phosphopeptide Enrichment Kits & Reagents	Thermo Scientific (Waltham, MA, USA)	Cat.#: A32992 & A32993
Pierce™ Quantitative Peptide Assays & Standards	Thermo Scientific (Waltham, MA, USA)	Cat.#: 23275

**Deposited data**		

Proteome Datasets	PRoteomics IDEntification Database (PRIDE)	PXD072701 (https://www.ebi.ac.uk/pride/archive/projects/PXD072701) https://doi.org/10.6019/PXD072701

**Experimental models: Organisms/strains**		

Mouse: C57BL/6J (7–8 weeks, male and female)	Jackson Labs (Waltham, MA, USA)	Strain #:000664 RRID: IMSR_JAX:000664

**Software and algorithms**		

Progenesis QI for proteomics (version 2.4)	Nonlinear Dynamics Ltd. (Newcastle Upon Tyne, UK)	https://www.nonlinear.com/progenesis/qi-for-proteomics/
Mascot (version 2.8)	Matrix Science (London, UK)	https://www.matrixscience.com/
Perseus	Tyanova et al.^7^ Tyanova & Cox^8^	https://maxquant.net/perseus/
g:Profiler	Kolberg et al.^9^ Kolberg et al.^10^	https://biit.cs.ut.ee/gprofiler/gost
DAVID	Huang et al.^11^ Sherman et al.^12^	https://davidbioinformatics.nih.gov/
SynGO	Koopmans et al.^13^	https://syngoportal.org/
iceLogo	Colaert et al.^14^	https://compomics.github.io/projects/iceLogo
pLogo	O'Shea et al.^15^	http://plogo.uconn.edu/
Xcalibur (version 4.7)	Thermo Scientific (Waltham, MA, USA)	Cat.#: OPTON-30967
Scaffold (version Scaffold_5.3.0)	Proteome Software (Portland, OR, USA)	https://www.proteomesoftware.com/
GraphPad Prism (version 10.4.2)	GraphPad Software (San Diego, California, USA)	https://www.graphpad.com/
R	R Core Team,^16^	https://www.r-project.org/

**Other**		

Mouse, Coronal (40–75g) Brain Matrix with 1 mm intervals	Ted Pella Inc. (Redding, CA, USA)	Cat.#: 15067
Fisherbrand Model 100 Ultrasonic Dismembrator with 1/8″ microtip probe (22.5 kHz converter)	Fisher Scientific (Pittsburgh, PA, USA)	Model#: XL2000-350R
Eppendorf Centrifuge 5804R with F-45–30–11 Rotor	Eppendorf (Hamburg, DE)	Cat.#: 022623508 Cat.#: 022637002
Centrivap Concentrator Vacuum Centri	LABCONCO (Kansas City, MO, USA)	Cat#: 7810000
Synergy 2 Microplate Reader	BioTek (Winooski, VT, USA)	Cat#: 7131000
Orbitrap Fusion Lumos Tribrid Mass Spectrometer	Thermo Scientific (Waltham, MA, USA)	Cat.# IQLAAEGAAPFADBMBHQ
EASY-Spray Source	Thermo Scientific (Waltham, MA, USA)	Cat.#: ES081
UltiMate 3000 RSLCnano System	Thermo Scientific (Waltham, MA, USA)	Cat.#: ULTIM3000RSLCNANO
EASY Spray C18 LC Column	Thermo Scientific (Waltham, MA, USA)	Cat.#: ES906
Solid Phase C18 ZipTip	MilliporeSigma (Billerica, MA, USA)	Cat.#: ZTC18S
LoBind® Polypropylene Tubes	Eppendorf (Hamburg, DE)	Cat.#: 022431021
Fisherbrand Model -FB-11203 Advanced Ultrasonic Cleaner	Fisher Scientific (Pittsburgh, PA, USA)	Cat.#: FB11203

## Materials and equipment

**Table:** Base RIPA Buffer

Reagent	Final concentration	Amount
Tris HCl (1.0 M)	20 mM	2 mL
NaCl (5.0 M)	150 mM	3 mL
EDTA (0.5 M)	2 mM	400 μL
SDS (10%)	0.1%	1 mL
H2O	N/A	Fill to 100 mL
**Total**	**N/A**	**100 mL**

Store Base RIPA buffer at 4°C for up to 12 months.

**Table:** RIPA-P Buffer with 1% Digitonin

Reagent	Final concentration	Amount	Category
Base RIPA Buffer	N/A	0.85 mL	Base RIPA Buffer
Triton X-100	1%	10 μL	Detergent
SDC (10%)	0.25%	25 μL	Detergent
NaV (150 mM)	1 mM	6.7 μL	Phosphatase Inhibitor
PMSF (200 mM)	1 mM	5 μL	Protease Inhibitor
NaF (200 mM)	1 mM	5 μL	Phosphatase Inhibitor
Protease Inhibitor Cocktail (100×)	1×	10 μL	Protease Inhibitor
Phosphatase Inhibitor Cocktail (100×)	1×	10 μL	Phosphatase Inhibitor
Digitonin	1%	10 mg	Detergent
**Total**	**N/A**	**1 mL**	–

Keep on ice until tissue homogenization step.

**CRITICAL:** Ensure inhibitors are fresh to prevent protein degradation and dephosphorylation during lysis.

***Alternatives:*** Other RIPA/lysis buffers may work for tissue homogenization. We found that the above RIPA buffer recipe worked best for our samples.

• Razor blades.
• 1 mm biopsy punch with ejector.
• Ice/dry ice/liquid nitrogen.
• Microcentrifuge tubes.

## Step-by-step method details

### Tissue collection and homogenization

**Timing: 3 h**

This section details the VTA tissue collection and homogenization steps needed to produce a soluble, quantifiable protein extract suitable for proteome-wide or phospho-enriched workflows.

**CRITICAL:** Practice and expertise are recommended, and training on spare animals is advised before an experimental cohort. Identification must be both rapid and consistent across animals.

1. Tissue Harvest (Figures 1 and 2).
  ***Note:*** Document the collection process and minimize the time between sacrifice and tissue freezing, keeping it as consistent as possible across samples. Delayed freezing compromises proteomic and phosphoproteomic analyses, as endogenous enzymes remain active and disrupt protein and phosphorylation levels. Additionally, minimize handling and stress of mice prior to collection, as these factors can alter the proteomic landscape of the target region.
  a. Extract the brain from the mouse and immediately place it into a chilled brain matrix.
    i. Ensure the brain is seated flat and fully in the matrix.
      ***Note:*** An unevenly seated brain will yield sections with misaligned landmarks.
  b. Prepare 1 mm thick coronal sections using the matrix (Figure 2, *top left*).
    i. Insert the first razor blade at the anterior aspect of the circle of Willis near the optic chiasm (AP: 0.02; Paxinos & Franklin, 2007, 3rd edition^17^).
    ii. Insert additional razor blades in 1 mm intervals along the matrix toward the posterior aspect of the brain.
    iii. Carefully remove the blades and separate them, keeping track of their orientation relative to the tissue, before placing them on ice. The VTA-containing section lies between the two red cuts shown in the mouse brain matrix (Figure 2, *top left*).
  c. Identify the VTA (AP: −2.80 to −3.88 mm; Paxinos & Franklin, 2007, 3rd edition) using stereotaxic coordinates and landmarks as target anatomy (Figure 2).
    ***Note:*** Atlas plates are a guide, not a template. They are spaced at intervals, so a slice may not match one plate exactly, and individual brains may differ from the atlas drawing. Use landmarks, not just coordinates, to visually confirm the VTA.
    i. Visually confirm the region is medial to the compact part of the substantia nigra excluding the medial lemniscus.
    ii. Using the plastic forceps, evaluate both faces of the slice to determine which tissue will yield the most VTA tissue.
      ***Note:*** The VTA is composed of multiple subregions (e.g., the parabrachial pigmented area (PBP), paranigral nucleus (PN), interfascicular nucleus (IF), and parainterfascicular nucleus (PIF)) that vary along the anterior-posterior axis. In the Paxinos & Franklin atlas, these subregions are labeled alongside the VTA and should not be mistaken for separate structures; the PBP designates the anterior portion of the VTA. A 1 mm punch spanning this AP window therefore pools tissue across these subregions rather than isolating a single subregion.
  d. Collect bilateral tissue samples using a 1 mm biopsy punch and place them into pre-chilled tubes on ice (∼1 mg/punch).
    ***Note:*** The 1 mm biopsy punch removes the sampled tissue and distorts the surrounding area. Punch placement is therefore guided by pre-punch anatomy and atlas-based target window shown in Figure 2. Punches may contain tissue from outside the VTA. To mitigate this, laser-guided microdissection or smaller punch sizes may be used but would require more mice.
  e. Snap-freeze the punched tissue immediately on dry ice or in liquid nitrogen.
  f. Clean collection tools to reduce risk of sample cross contamination and repeat collection steps (1a-f) for each animal.
  g. Store samples at −80°C or in liquid nitrogen until they are ready to process.
    ***Note:*** Pool 2 bilateral punches per sample for proteomics and 3 for phosphoproteomics.
    More tissue would be ideal for phosphoproteomic analyses. This, however, may be difficult because of the small size of the brain region. Newer mass spectrometers offer improved depth of proteome coverage with less starting material and would be an optimal alternative for this workflow.
    Before running full cohorts of experiments, optimize the ideal protein range of samples for your equipment and tissue constraints while using the smallest feasible number of mice per sample.workflow.
    **Pause point:** (Snap-frozen tissue samples may be stored at −80°C but should be processed as soon as possible (recommended within ∼3 months). Prolonged freezing may alter subsets of the proteome and phosphoproteome.^18^)

**Figure 1:**
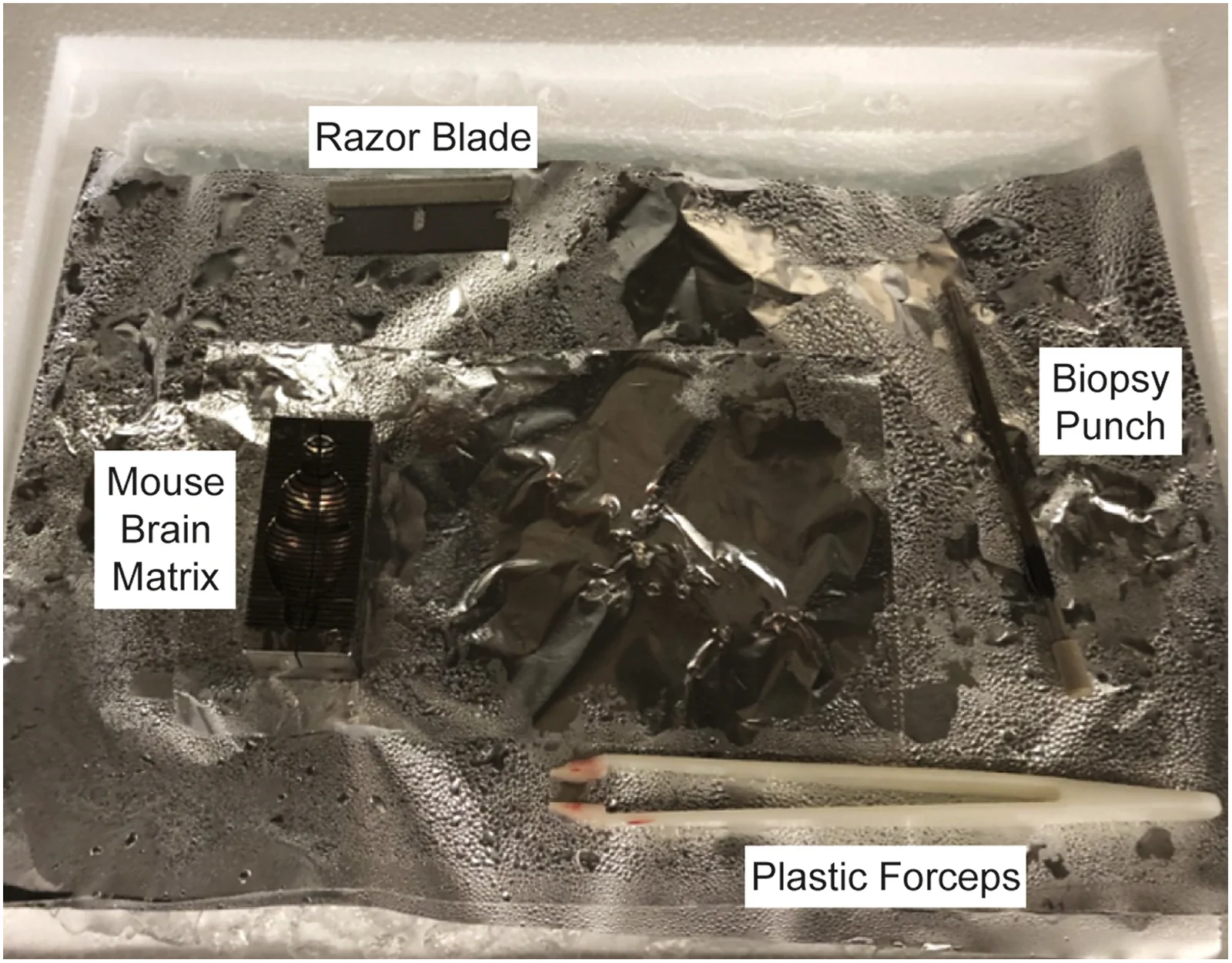
Tissue Collection Setup Tissue collection setup on an ice-cold surface. Tools used for VTA dissection: 1 mm biopsy punch with ejector, razor blade, chilled brain matrix, plastic forceps, and Styrofoam container filled with ice and covered with aluminum foil.

**Figure 2:**
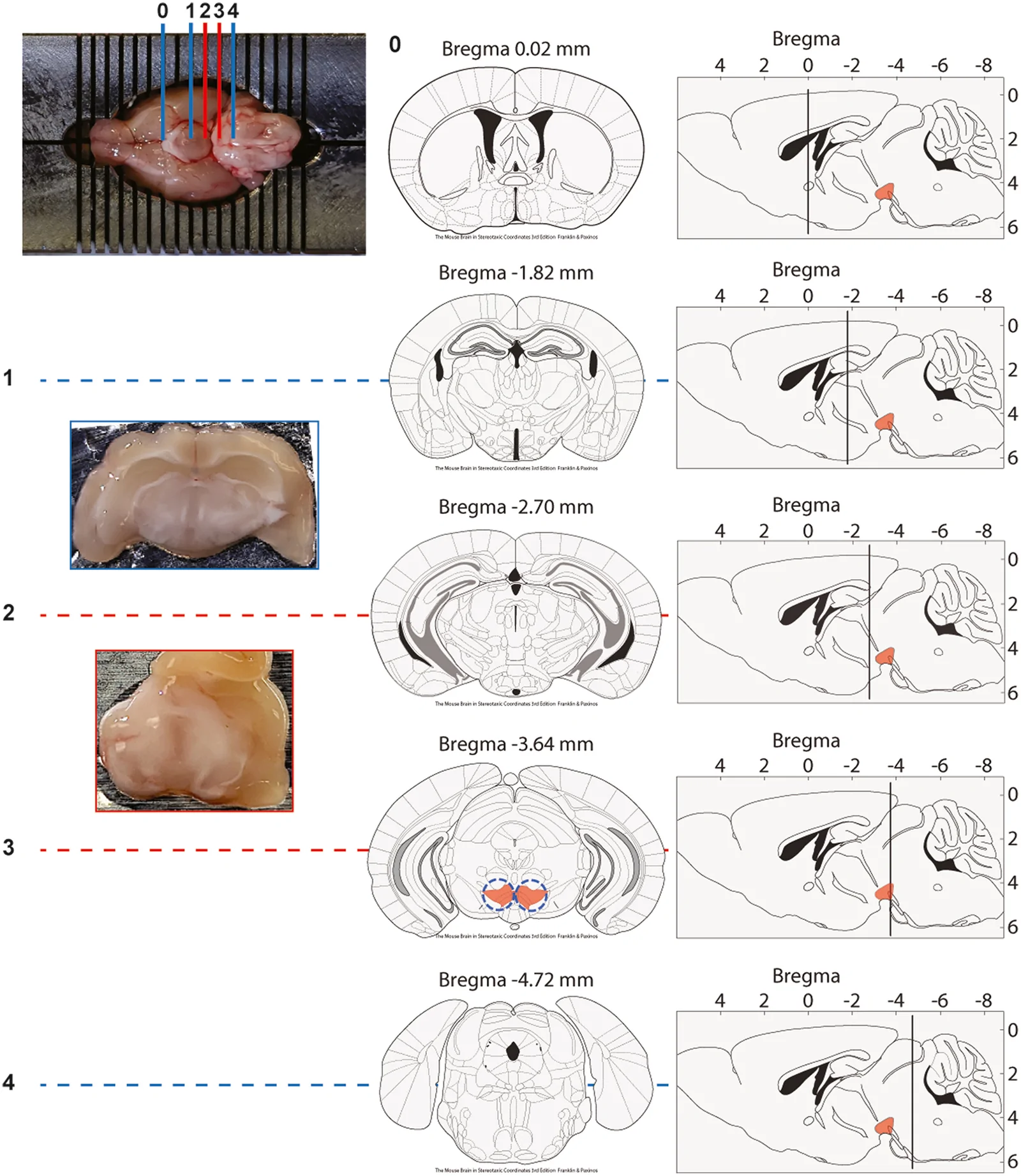
Stereotaxic localization of the mouse VTA for biopsy-punch collection Guide for the sectioning and identification steps of tissue harvest (Steps 1a-d), using the Paxinos & Franklin mouse brain atlas (2007, 3rd edition). *Top left*: ventral view of a mouse brain seated in the chilled coronal matrix, exposing the circle of Willis and optic chiasm landmarks used to place the first cut (Step 1b.i), with five blade positions marked (blue: 0, 1, 4; red: 2, 3). Each blade position has a corresponding row (0–4) pairing the coronal atlas plate (*center*) with a sagittal schematic (*right*) showing the VTA—including its component subregions—in orange and the plane of section as a vertical black line. Coordinates shown are from a representative dissection; in practice, cut faces fall at slightly different levels across animals. Sectioning begins at the anterior aspect of the circle of Willis (Row 0, AP: 0.02 mm) and proceeds posteriorly in 1 mm intervals starting at Row 1 (AP: −1.82 mm). The VTA-containing section lies between the two red cuts (Rows 2–3): the anterior cut (Row 2, AP: −2.70 mm) falls anterior to the VTA’s atlas-defined boundary (red-outlined tissue section at left) while the posterior cut (Row 3, AP: −3.64 mm) passes through the posterior aspect of the VTA (coronal plate: VTA in orange, blue dashed circles indicating bilateral 1 mm punch target window). Slice faces routinely fall between atlas plates and individual brains vary from the atlas drawing. The atlas therefore serves as a starting framework, and the harvested section is confirmed by landmarks relative to adjacent plates. Row 1 (AP: −1.82 mm, blue-outlined tissue section at the *left*) shows sectioning anterior to the VTA and row 4 (AP: −4.72 mm) lies posterior to the VTA. Tissue photographs were cropped and adjusted for exposure, brightness, contrast, saturation, sharpness, and color temperature, with denoising applied, to improve visibility of anatomical detail. Adjustments were applied to the entire image.
2. Tissue Lysis.
  a. Transfer pooled frozen tissue samples to a chilled tube on ice.
  b. Add 10 to 15 μL supplemented RIPA buffer (containing inhibitors and 1% digitonin) per punch for proteomic analysis, and 75 to 80 μL per punch for phosphoproteomic analysis.
3. Homogenization and Protein Collection (Figure 3).
  a. Homogenize the tissue-buffer mixture on ice using the probe of a sonic dismembrator.
    i. Clean the probe before sonication.
    ii. Set the power control knob (continuous 1–10 scale) on the Fisherbrand Model 100 ultrasonic dismembrator with 1/8″ microtip probe to six.
      ***Note:*** Because output is instrument- and probe-dependent, the numerical power settings may not transfer directly between systems. Users should optimize an equivalent condition that achieves complete homogenization (Figure 3) without sample heating.
    iii. Sonicate in very short bursts (∼2 seconds) with 2-s breaks between until the sample is fully homogenized (Figure 3).
    iv. After homogenization is complete, clean the probe and repeat steps 3a.i–iii for all samples.
      **CRITICAL:** Sonicate in very short bursts to prevent overheating the sample. Overheating can interfere with protease and phosphatase inhibitor function.
      ***Alternatives:*** Other homogenization methods may be used for this step. Bead beating is an excellent alternative for mass-spec workflows, as it can simultaneously lyse multiple samples in a highly efficient and reproducible manner. Because each method has advantages and disadvantages, investigators should carefully select the approach best suited for their intended analyses.
  b. Incubate the samples on ice for 15 min.
  c. Centrifuge the homogenate at 15,000 × *g* for 10 min at 4°C.
  d. Carefully transfer the supernatant to a new pre-chilled tube, avoiding the pellet.

**Figure 3:**
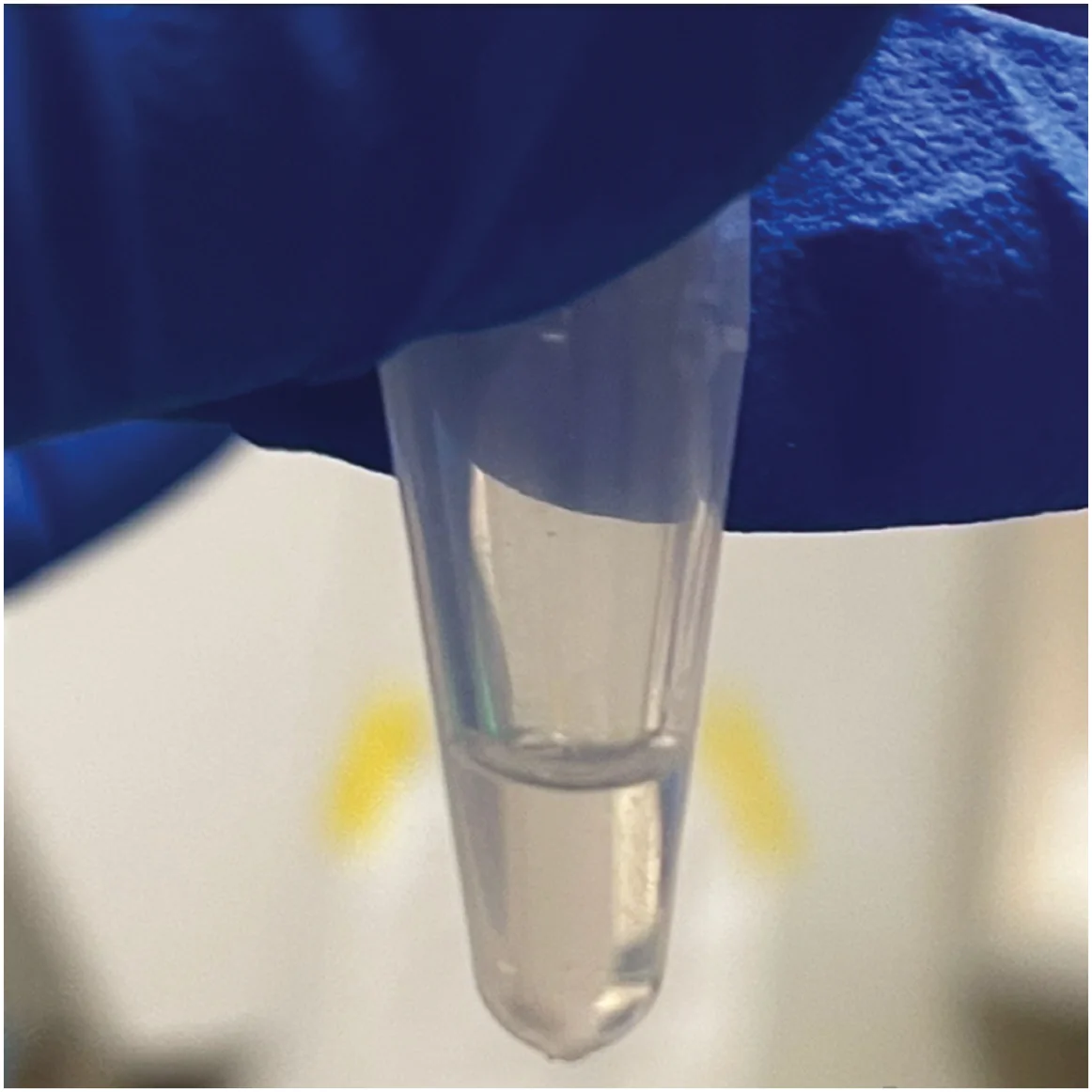
Representative VTA homogenate after sonication Clear homogenate containing solubilized VTA protein following sonication, prior to centrifugation. A clear, uniform solution free of visible tissue fragments indicates complete disruption of the pooled punches; cloudiness or visible particulates indicate incomplete lysis, in which case sonication should be extended.
4. Protein Quantification.
  a. Determine the protein concentration of the supernatant using the Pierce BCA Protein Assay Kit according to the manufacturer’s instructions (https://documents.thermofisher.com/TFS-Assets/LSG/manuals/MAN0011430_Pierce_BCA_Protein_Asy_UG.pdf).
5. Place sample in −80°C until MS sample preparation steps.

**Pause point:** (Protein lysates may be stored at −80°C for up to 6 months before MS sample preparation. Ideally samples should be used as soon as possible; aliquoting is recommended to avoid multiple freeze-thaw cycles.)

### In-gel digestion and peptide purification (proteome)

**Timing: 2 days**

The following section outlines the procedure for fractionating 50 μg of VTA lysate (approximately 1.5 to 2.5 μg/μL) by SDS-PAGE, digesting gel slices with trypsin, and purifying peptides on C18 ZipTips to generate MS-ready samples for label-free proteome-wide quantification.

6. Gel electrophoresis.
  a. Load 50 μg of the VTA protein lysate onto a 10% SDS-PAGE gel.
  b. Run the gel until the dye front has migrated sufficiently (∼0.5 cm away from the bottom of the gel).
  c. Partition each lane into seven horizontal slices.
  d. Cut each gel slice into 1–2 mm cubes.
  e. Place the gel cubes from each slice into a separate 0.6 mL LoBind tube.

***Note:*** Each gel lane was divided into seven fractions, representing the balance used in our workflow between proteome depth, LC-MS/MS acquisition time, and supply costs. Greater fractionation may increase analytical depth but also increases sample-processing and instrument time.

7. Destaining and dehydration.
  a. Add 375 μL of the destaining solution (50% acetonitrile (ACN) in 40 mM NH_4_ HCO_3_) and shake for 15 min at 600 rpm and 37°C.
  b. Tap tubes vigorously by hand (∼3 firm taps).
  c. Shake an extra 10 min @ 600 rpm, 37°C.
  d. Remove the destaining solution and add 100 μL 100% ACN to dehydrate the gel pieces for 15 min at 20–25°C with no shaking.
  e. Aspirate the ACN and dry the gel pieces in a vacuum centrifuge at 60°C for 30 min.
8. Trypsin digestion.
  a. Rehydrate the dried gel pieces by adding 20 μL of trypsin solution (250 ng trypsin in 40 mM NH_4_ HCO_3_).
  b. Incubate the tubes at 4°C for 15 min to allow for trypsin absorption.
  c. Add an additional 50–100 μL of 40 mM NH_4_ HCO_3_ to cover the gel pieces (avoid introducing bubbles into the liquid).
  d. Incubate the tubes with gentle shaking (300 rpm) for 14 to 20 h at 37°C.
  e. Terminate digestion by adding 10 μL of 5% formic acid (FA).
  f. Incubate at 37°C for 30 min, then centrifuge for 1 min.
9. Peptide extraction.
  a. Transfer the supernatant to a clean LoBind polypropylene tube.
  b. Perform a second extraction on the remaining gel pieces using 40 μL of 0.5% FA.
  c. Combine the supernatants from both extractions.
  d. Dry the combined extracts in a vacuum centrifuge until the volume is reduced to approximately 5–10 μL.
10. C18 purification (ZipTip).
  a. Add 10 μL of loading buffer (0.05% heptafluorobutyric acid (HFBA)/5% FA) to the dried sample and incubate at 20–25°C for 15 min.
  b. Pre-wet the solid-phase C18 ZipTip with 20 μL of ACN, avoid drawing air into the C18 tip.
  c. Load the peptide mixture onto a solid-phase C18 ZipTip by pulling the sample through the tip back and forth 10 times, again, avoid drawing air into the C18 tip.
  d. Wash the ZipTip with 35 μL of wash buffer (0.005% HFBA/5% FA).
  e. Elute peptides sequentially with 4 μL of 50% ACN/1% FA followed by 4 μL of 80% ACN/1% FA (pull the sample through the tip back and forth 10 times per elution).
  f. Combine the eluates and dry them completely using a vacuum centrifuge.
  g. Resuspend the final dried peptides in 6 μL of 0.1% FA and sonicate for 2 min.
  h. Transfer 3.0 μL of the final sample to an autosampler vial.
11. System setup.
  a. Verify that the Orbitrap Fusion Lumos tribrid mass spectrometer is calibrated.

***Note:*** Ensure that the column is fit for use in chromatographic alignment-based experiments by using a column that is either new or has not been used for many samples. Consider running a HeLa digest (ThermoFisher Scientific, Cat.# 88329) with the instrument method to ensure proper yields at both the protein and peptide level, consistent with benchmark numbers.

Acceptable performance is defined by total peptide/protein identifications and chromatographic consistency relative to the laboratory’s established HeLa baseline for that instrument. A decrease of approximately 10% or more from baseline should prompt system maintenance (cleaning, calibration verification, column inspection) before sample analysis. Because absolute HeLa performance is equipment-dependent, investigators should establish a local baseline with the same LC-MS/MS method.

12. Gradient and acquisition.
  a. Inject 2.5 μL of the prepared sample onto the column.
  b. Buffer A = 100% H2O in 0.1% FA; Buffer B = 80% ACN in 0.1% FA.
  c. Execute the gradient:

**Table:** 

Gradient step	Time (min)
Loading on trap column	0–15 (15 min) (flow rate 0.300 μL/min)
Linear gradient (1%–35% B)	16–135 (119 min) (flow rate 0.220 μL/min)
Step to 99% B	135–139 (5 min) (flow rate 0.220 μL/min)
5 min Hold	139–144 (5 min) (flow rate 0.250 μL/min)
Step to 1% B	144–149 (1 min) (flow rate 0.250 μL/min)
10 min Hold	149–159 (10 min) (flow rate 0.250 μL/min)
  d. Operate the Orbitrap Fusion Lumos using Xcalibur with the following parameters:

**Table:** 

Parameter	Value
Ion mode	Positive
Full MS resolution (Orbitrap)	120,000
Scan range	m/z 275–1475
AGC target (Full MS)	4 × 10^5^
Max Injection time	Auto
MS/MS method	TopSpeed, HCD
Isolation window	2.0 Th
Collision energy	35% HCD
Dynamic exclusion	60 s, repeat count 1
Cycle Time	3s
13. Label-free quantification.
  a. Import.raw files into Progenesis QI (v2.4) and convert them into total ion chromatograms (retention time vs. m/z). (Figure 4A).
    ***Note:*** If there are issues importing.raw files into Progenesis QI (or alternative software of choice), check for issues with the file and acquisition (e.g., file size, error logs, etc.)
    **CRITICAL:** Always keep original.raw files.

**Figure 4:**
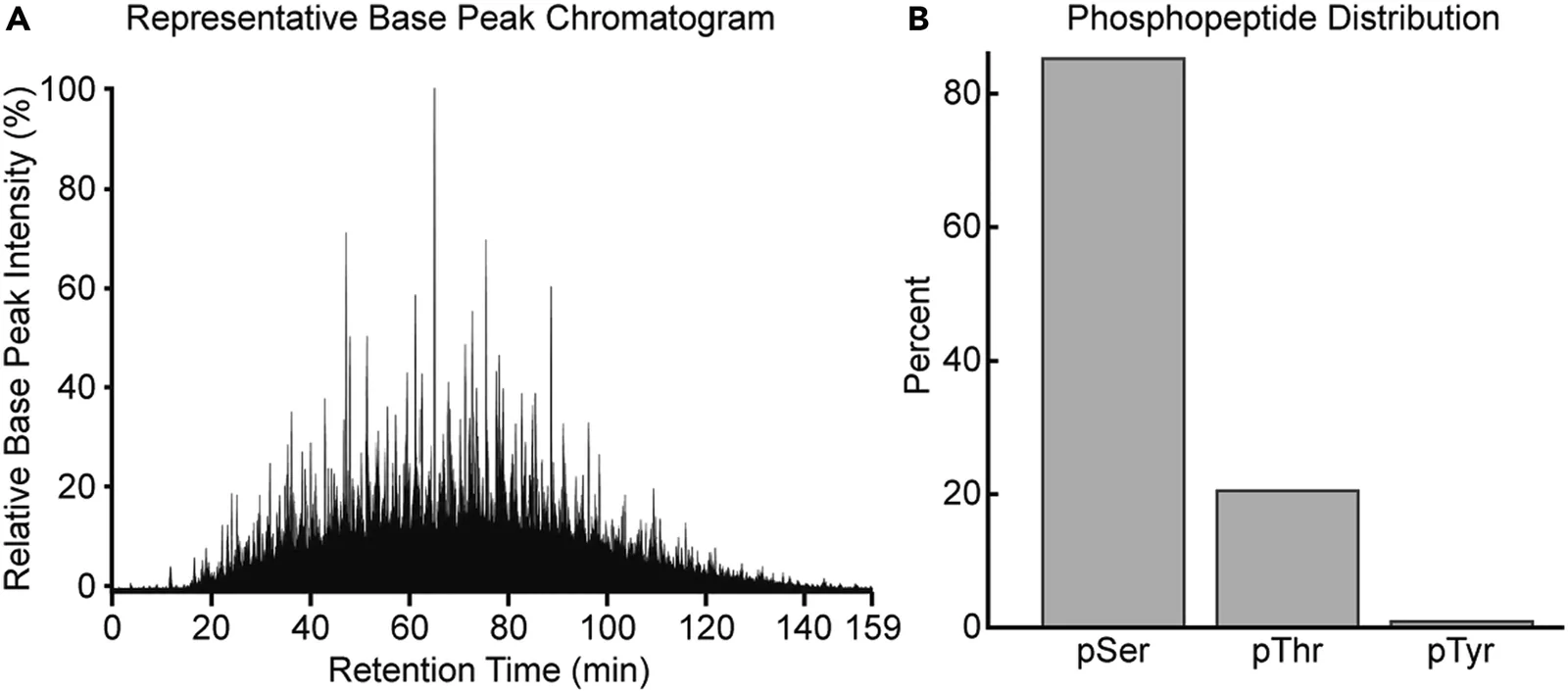
Representative LC-MS/MS chromatogram and phosphopeptide distribution (A) Representative base-peak chromatogram from a nano-LC-MS/MS run of a VTA sample, showing peptide elution peaks across the reversed-phase gradient. The x-axis is retention time; the y-axis is ion intensity (relative scale). (B) Phospho-amino-acid distribution following SMOAC enrichment. Bar graph showing the relative proportion of phosphoserine (pSer), phosphothreonine (pThr), and phosphotyrosine (pTyr) identified using an Orbitrap Fusion Lumos Tribrid mass spectrometer. The distribution is dominated by pSer, consistent with expected eukaryotic phosphoproteome.
  b. Go through the automatic selection of a reference run and alignment steps.
  c. For the resulting aggregate dataset, filter for +2, +3, and +4 charged ions.
  d. Exclude MS/MS spectra with rank > 10 and export the peak list (.mgf).
  e. Search the.mgf file against the most recent *Mus musculus* SwissProt database using Mascot.
  f. Set the following search parameters:
    i. 10 ppm precursor tolerance.
    ii. 0.5 Da product tolerance.
    iii. Trypsin digestion with up to 2 missed cleavages.
    iv. Variable modifications of methionine oxidation, lysine acetylation, and phosphorylation of S/T/Y, ^13^C = 1.
  g. Import results to Progenesis, exclude peptides with Mascot Ion Score < 25, and normalize peptide ion abundance to all proteins.
  h. Filter the resulting peptide and protein identifications to retain only those proteins identified by at least two unique peptides and peptide sequence confidence score ≥ 95%, and protein identification confidence of ≥ 99% to ensure high-confidence protein quantification.
    ***Note:*** Peptide-ion abundance is normalized per run in Progenesis against an automatically selected reference run (the run least different from the dataset) using the mean of the log abundance ratios of peptide ions, excluding outlier ions; this is a ratio-based scaling to a common reference, not a total-ion-current normalization. Because Progenesis aligns retention time and quantifies features by co-detection on an aggregate map, a feature detected in any run is measured across all runs, minimizing missing values. Therefore, no missing-value imputation is performed. The few features not quantified across runs are excluded rather than imputed, and mixed-effects models are used for repeated-measures comparisons where residual missing values occur.

### Phosphoproteomic workflow

**Timing: 2 days**

The following steps describe the phosphoproteomic preparation including digesting 200–400 μg of the VTA lysate (approximately 0.5 to 1.3 μg/μL) in solution, enriching phosphopeptides by SMOAC for enhanced phosphopeptide identification, and resuspending for LC-MS/MS injection.

14. In-solution tryptic digestion.
  a. Aliquot 200–400 μg of VTA protein lysate.
  b. Precipitation.
    i. Add 4 volumes of −20°C 100% acetone to each sample.
    ii. Vortex and incubate at −20°C for 1 to 20 h to precipitate proteins.
    iii. Centrifuge at 18,000 × *g* for 10 min at 4°C.
    iv. Carefully decant the acetone without dislodging the pellet(s).
    v. Remove excess acetone by carefully tapping tube(s) onto a Kimwipe.
    vi. Add 400 μL of −20°C 90% acetone and vortex.
    vii. Repeat steps 14b.iii to 14b.v.
    viii. Allow pellet(s) to air-dry for 3 min.
  c. Resuspension.
    i. Add 100 μL of Digestion Buffer (50 mM NH4HCO3, 1% SDC) and resuspend by pipetting up and down to break the pellet. (Adjust volume if necessary).
    ii. Ensure complete dissolution of protein by using the bath sonicator for 5 min.
      **Table:** Bath Sonicator Settings

Mode	Sweep
**Freq**	37 kHz
**Power**	100
      ***Note:*** If needed add another 5-minute cycle.
    iii. Spin sample(s) down in centrifuge.
  d. Reduction.
    i. Add 200 μL of ultrapure water to a No-Weigh DTT tube (Thermo Scientific, Cat.# 20290) and pipette up and down until dissolved (final concentration is 250 mM).
    ii. Add 2.0 μL of 250 mM DTT to each sample (final concentration is 5 mM).
    iii. Vortex for several seconds on low setting (3–4) and incubate at 56°C for 30 min.
    iv. Cool sample(s) to 20–25°C for 10 min.
  e. Alkylation.
    i. Add 1.5 μL of 1.0 M acrylamide to each sample (final concentration is 15 mM).
    ii. Vortex for several seconds on low setting (3–4) and incubate at RT for 30 min protected from light.
    iii. Add 2.0 μL of 250 mM DTT to each sample to quench the alkylation reaction. Vortex on low setting and incubate in the dark at RT for 15 min.
  f. Digestion.
    i. Add stock Lys-C to each sample at 1 μg of enzyme to 100 μg of substrate. (ThermoFisher, Cat.#: 90307).
      ***Note:*** If using less or more protein, decrease or increase the amount of Lys-C appropriately to maintain an Enzyme:Substrate = 1:100.
    ii. Incubate on shaker for 2 h at 300 rpm, 37°C.
    iii. Add 50 μL of 50 mM NH4HCO3.
    iv. Add stock trypsin (ThermoFisher, Cat.#: 90057) to each sample at 1 μg of enzyme to 50 μg of substrate.
      ***Note:*** If using less or more protein, decrease or increase the amount of trypsin appropriately to maintain an Enzyme:Substrate = 1:50.
    v. Incubate for 14 to 20 h on shaker at 300 rpm, 37°C.
    vi. Add 14.7 μL of 40% FA/1% HFBA to each sample to get final concentration of 4% FA/0.1% HFBA and mix gently by pipetting.
      ***Note:*** Acidification simultaneously stops trypsin digestion and precipitates SDC from digestion buffer.
    vii. Let sample(s) sit for 10 min at RT.
  g. Dry down samples to zero volume.
15. Phosphopeptide enrichment.
  a. Enrich phosphopeptides using a sequential enrichment metal oxide affinity chromatography (SMOAC) kit (Thermo Scientific, Cat.#: A32992 & A32993), following the manufacturer’s protocol. (https://www.thermofisher.com/blog/learning-at-the-bench/wp-content/uploads/sites/13/2024/10/High-SelectTM-SMOAC-Protocol.pdf).
    i. Equilibrate a High-Select TiO_2_ spin tip (Thermo Scientific, Cat.# A32993) with Wash Buffer and Binding/Equilibration Buffer.
    ii. Load the peptide sample, centrifuge, and reapply the flow-through. Retain the flow-through.
    iii. Wash the tip with Binding/Equilibration Buffer, Wash Buffer, and LC-MS-grade water.
    iv. Elute bound phosphopeptides with Phosphopeptide Elution Buffer and dry immediately.
    v. Combine the TiO_2_ flow-through and wash fractions, dry, and resuspend in Fe- NTA Binding/Wash Buffer.
    vi. Equilibrate a High-Select Fe-NTA spin column (Thermo Scientific, Cat.# A32992) and load the resuspended sample. Incubate for 30 min with gentle mixing.
    vii. Wash the Fe-NTA column with Binding/Wash Buffer and LC-MS grade water.
    viii. Elute phosphopeptides with Elution Buffer and dry immediately.
    ix. Resuspend both eluates in 0.1% FA for peptide quantification and LC-MS/MS analysis.

***Note:*** Following tryptic digestion and SMOAC enrichment, phosphopeptides are typically recovered at ∼1%–3% of the starting sample load (determined using the Pierce Quantitative Colorimetric Peptide Assay Kit, Step 16b) and comprised ∼75% of the total unique peptide sequences under the conditions used here.

16. MS sample preparation.
  a. Resuspend the dried phosphopeptides in 20 μL of 0.1% FA (v/v).
  b. Quantify peptide concentration using the Pierce Quantitative Colorimetric Peptide Assays & Standards Kit (Thermo Scientific, Cat.# 23275) per the manufacturer’s instructions (https://documents.thermofisher.com/TFS-Assets/LSG/manuals/23275_quantpeptide_color_UG.pdf).
    i. Rehydrate the Peptide Digest Assay Standard (1 mg/mL) with 1.5 mL of 50 mM ammonium bicarbonate to create a 1,000 μg/mL stock. Prepare serial standards by diluting in 50 mM ammonium bicarbonate (range: 1,000 → 15.6 μg/mL).
    ii. Prepare the Working Reagent by mixing 50 parts Reagent A + 48 parts Reagent B + 2 parts Reagent C (e.g., 5 mL + 4.8 mL + 0.2 mL per plate).
    iii. Add 20 μL of each standard or unknown sample to separate wells of a 96-well plate.
    iv. Add 180 μL of Working Reagent to each well.
    v. Mix on a plate shaker for 30 seconds.
    vi. Incubate at 22°C for 30 min (or 37°C for 15 min).
    vii. Read absorbance at 480 nm.
    viii. Generate a standard curve from the peptide standards and calculate the peptide concentration of the unknown samples.
    ix. Prepare 350 ng of the purified sample for mass spectrometry.
17. For mass spectrometry, follow the same procedure as the above proteomic mass spectrometry steps (steps 11 to 13) with 350–800 ng of the purified sample.

***Note:*** Once the data processing is complete in Progenesis, export a peptide ion measurements list and evaluate the quality of your phosphopeptide enrichment by extracting the total number of unique spectra, then deduplicating the list to obtain the total number of unique peptide sequences. Next determine the percentage of those peptide sequences that contain a phospho modification.

### Integrated downstream bioinformatics and functional enrichment analysis

**Timing: Variable**

This section outlines the step taken in the associated study^1^ to convert normalized MS feature tables into biological insight by visualizing sample-to-sample structure and testing the resulting protein lists for GO enrichment (broad and synapse-specific) and phosphosite kinase motif inference.

18. Statistical handling of MS-derived features.
  a. Analyze both proteomic and phosphoproteomic datasets in Progenesis QI using built-in label-free quantification framework.
  b. Predefine statistical significance as appropriate.
    ***Note:*** The associated study^1^ used a nominal *p*-value < 0.05 for exploratory discovery. For prospective analyses using this protocol, Benjamini-Hochberg FDR correction (*q* < 0.05) is recommended to reduce false positives from proteome-wide measurement. The choice between using nominal *p*-value and further FDR correction depends on the experimental question and design. Nominal *p*-values may be used alone when the experimental goal is to retain all candidate proteins that pass the initial ANOVA, without a rank-based filter that judges each protein relative to the rest of the dataset. This matters when measured changes are small or proteins are low in abundance, such as subtle shifts of localized responses diluted within heterogenous tissue, as the FDR correction may filter these out along with false positives. Findings from either method are hypothesis-generating and require subsequent validation.
  c. Apply ANOVA statistical analysis across experimental groups to identify differentially expressed proteins (DEPs); use mixed-effects models where missing values or specific repeated measures occur.
    i. Apply post-hoc tests appropriate to the experimental design. Report the specific test used and the multiple-testing correction method, if used.
  d. Export the filtered peptide/protein table as a comma-separated (.csv) or tab-separated (.txt) file, retaining the following columns: Protein Accession, Description, Abundance (one column per sample), p-value, and fold change.
  e. Import the exported file into Perseus,^7^^,^^8^ or similar program.
  f. Apply Z-score normalization to the filtered datasets in Perseus using the built-in Z-score transformation.
    ***Note:*** This Z-score transformation converts the protein abundances (proteomic) and peptide ion intensities (phosphoproteomic) and is applied for visualization (PCA and heatmaps) to make differences comparable across runs; it does not replace or re-normalize the Progenesis abundance normalization described in step 13, which is the normalization used for all statistical testing.
19. PCA and sample clustering.
  a. Run principal component analysis (PCA) in Perseus using retained normalized features and plot samples along PC1 (largest variance) and PC2 (second-largest variance) to assess overall sample variance, replicate consistency, potential outliers and technical or biological structure.

**Figure 5:**
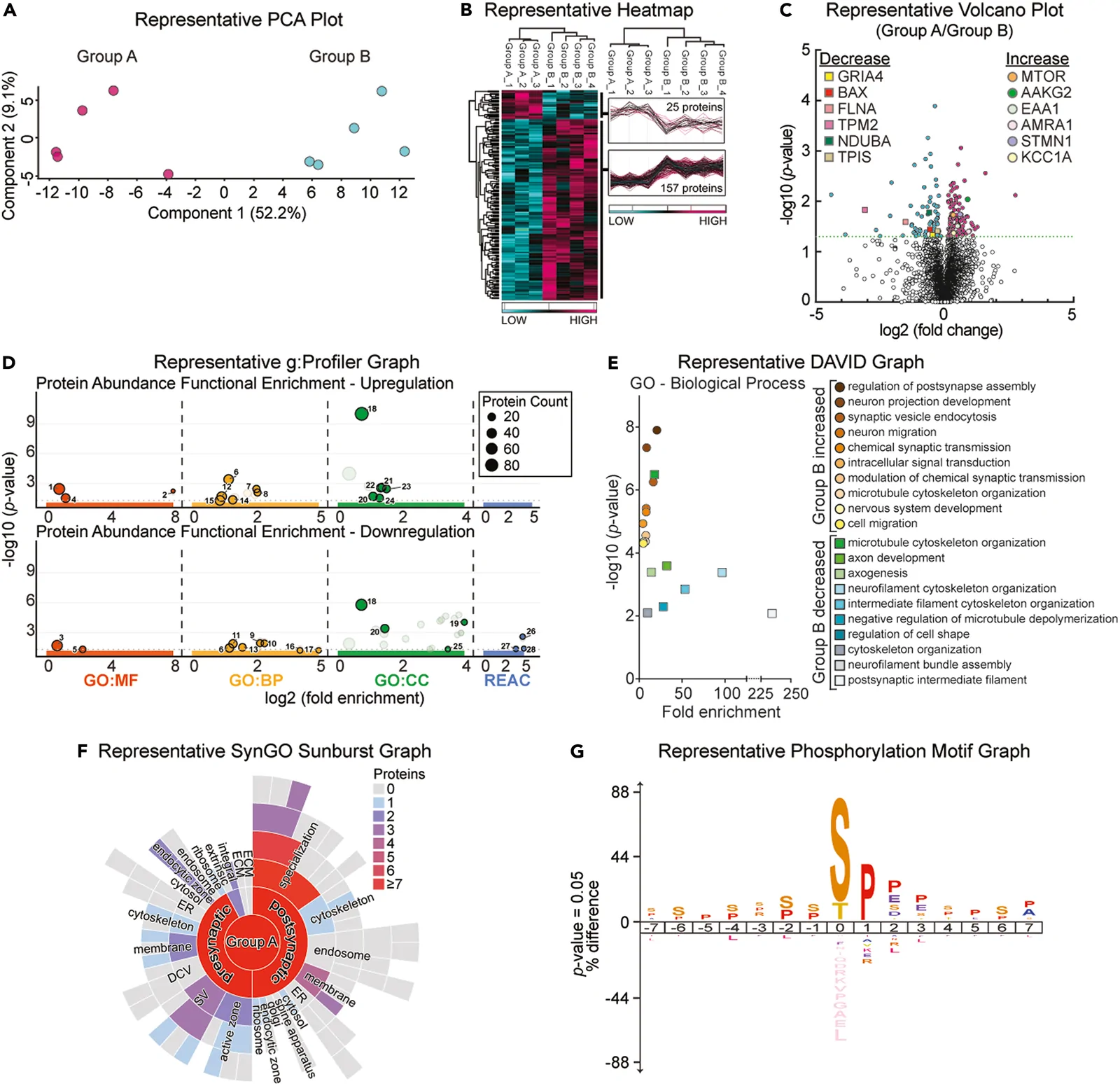
Representative proteomic/phosphoproteomic graphs (A) Principal component analysis (PCA) plot of significantly different Z-scored protein or phosphopeptide abundances. Samples are colored by experimental group. Each axis is labeled with the principal component and the percent variance it explains (e.g., PC1 = 52.2%). (B) Hierarchical clustering heatmap of differentially expressed proteins/phosphopeptides (rows) across samples (columns), using Euclidean distance and z-score normalization. The color scale (red to blue) indicates high to low relative abundance after z-score transformation; the dendrograms show sample and feature similarity. (C) Volcano plot of differentially expressed proteins/phosphopeptides. The x-axis is log2 (fold change) and the y-axis is -log10 (p-value). Differentially expressed features were defined as p < 0.05 (two-tailed *t* test). Colored points indicate up-regulated and down-regulated features relative to the comparison group; gray points are not significant. (D) g:Profiler functional-enrichment dot plot showing GO and Reactome terms for up- and down-regulated proteins. The x-axis is log2 (fold enrichment) by enrichment categories and the y-axis shows -log10 (p-value) of enrichment terms. Dot size reflects the number of proteins in each term. Dot opacity reflects g:Profiler-identified driver terms, where solid dots represent significant driver terms and translucent dots represent significant non-driver enrichments. (E) DAVID functional-annotation plot of GO biological process terms ranked by fold enrichment and p-value. The x-axis is fold enrichment and the y-axis lists GO biological process terms. Significance threshold: *p* < 0.05. (F) SynGO sunburst plot showing synapse-specific localizations. Concentric rings represent GO and SynGO term hierarchy. Colored sectors indicate synaptic compartments or processes and their relative protein contribution. (G) iceLogo/pLogo phosphorylation motif generated from peptide sequences centered on differentially expressed phosphosites (central residue at position 0; ±7 flanking residues). The y-axis shows the statistical significance of amino acid overrepresentation at each position. Residues above the baseline are significantly enriched relative to the total phosphoproteome distribution. All graphs adapted with permission from Diezel et al.^1^

***Note:*** The associated study used PCA restricted to significantly altered features as a post-selection visualization of differential patterns, reflecting the study’s aim of visualizing whether a coordinated pattern of significantly altered protein abundance was present within the VTA samples. For prospective assessment of overall sample variance, replicate consistency, technical structure and potential outliers, PCA using all retained normalized features is recommended. In this application, the highest-variance features reflect sample quality, and the analysis is limited to quality assessment rather than a test of group differences. Experimental-group separation is not required for a technically valid PCA, and its absence does not rule out robust differential changes. PCA restricted to differential features may additionally be used as an exploratory visualization (Figure 5A) but should be identified as a post-selection analysis, since selecting features based on between-group differences guarantees some separation by construction, and the result depends on the significance threshold used for selection.

20. Perform hierarchical clustering using Euclidean distance to generate a heatmap of DEPs across samples to visualize sample-to-sample similarity using normalized Z-scores (Figure 5B).
21. Visualize DEPs and differentially expressed phosphopeptides as volcano plots by their log2 (fold change) on the x-axis and –log10 (*p*-value) on the y-axis using the statistical software of your choice (Figure 5C).
  a. Add a reference line at the predetermined *p*-value (i.e., *p* < 0.05).

***Note:*** For visualization purposes, the *p*-value is converted to the -log10 (*p*-value).

***Note:*** To be more conservative, establish a predefined fold-change threshold (e.g., |log_**2**_ FC| > 0.5) before analyzing the data. This reduces noise but may also exclude biologically meaningful hits with moderate changes.

22. Functional annotation and pathway enrichment.
  ***Note:*** The downstream analyses described here use established bioinformatic resources. The purpose of this section is to define the sequence, inputs, parameters, and interpretation used in the associated workflow rather than to introduce new computational software.
  **CRITICAL:** g:Profiler and SynGO require gene symbols and will not accept protein accession or database IDs. Conversion must be done prior to enrichment submission. This conversion can be done using various tools like those available through DAVID or UniProt.
  a. Use g:Profiler^9^^,^^10^ for Gene Ontology (GO) and Reactome pathway enrichment analyses to capture “driver” GO enrichment terms (Figure 5D).
    i. Use the gprofiler2 package or the g:Profiler website (https://biit.cs.ut.ee/gprofiler/gost) for enrichment queries.
    ii. Convert differentially expressed protein (DEP) names to accepted gene names.
    iii. Submit the converted list as the input query.
    iv. Set organism to *Mus musculus,* apply the g:SCS algorithm to adjust for multiple testing, and use *p* < 0.05 as a significance threshold.
    v. Select the following annotation databases: GO Biological Process, GO Molecular Function, GO Cellular Component, and Reactome.
    vi. In the output, identify “driver terms”.
    vii. For visualization, download bubble plots generated from the g:Profiler website or generate your own through R^16^ using gprofiler2 results and R packages like ggplot2. Plot the -log10 (adjusted *p*-value) on the y-axis with log2 (fold enrichment) on the x-axis.
      ***Note:*** Driver terms are statistically overrepresented parent GO terms that summarize clusters of related child terms, reducing redundancy in the enrichment results.
  b. Use the DAVID tool^11^^,^^12^ as a complementary annotation resource for GO and REACTOME pathway terms (Figure 5E).
    i. Submit the same list used for g:Profiler to the DAVID tool (https://davidbioinformatics.nih.gov/).
    ii. Focus on GOTERM_BP_DIRECT, GOTERM_CC_DIRECT, GOTERM_MF_DIRECT, and Reactome pathways.
    iii. Plot the top 10 terms by their *p*-value and fold enrichment (e.g., as a scatter plot or another appropriate method).
      ***Note:*** Unlike g:Profiler, DAVID does not provide driver terms. Due to this and the number of terms identified through DAVID, only the top 10 to 15 significant terms should be plotted to avoid overwhelming this plot.
  c. Use SynGO^13^ to restrict enrichment analysis to synapse-specific subcellular localizations and processes (presynaptic, postsynaptic, synaptic organizers, etc.) (Figure 5F).
    i. Convert the mouse gene symbols used for g:Profiler to their human ortholog gene symbols or HGNC gene IDs.
    ii. Submit the converted list to SynGO (https://syngoportal.org/) with the following parameters: “brain expressed” background, minimum of 3 genes per GO term, most recent SynGO release.
    iii. Customize the sunburst plot to best represent your data and download it.
      ***Note:*** SynGO is curated for synaptic proteins. Non-synaptic proteins may not appear in SynGO output. After downloading the sunburst plot, further stylistic adjustment can be made.
23. For phosphorylation motif analysis, use tools such as iceLogo^14^ or pLogo.^15^ (Figure 5G).
  ***Note:*** For phosphopeptides with multiple candidate S/T/Y residues, phosphosites are assigned by Mascot (step 13). The Ion Score ≥ 25 filter ensures confident peptide identification, but this workflow and the associated study did not score phosphosite localization confidence. Where stricter site assignment is desired, phosphosite localization confidence scores can be calculated post hoc (e.g., Mascot Delta Score, Ascore, phosphoRS/ptmRS, etc.) and only high-confidence sites (e.g., probability ≥ 0.75, AScore ≥ 13, etc.) retained before alignment, since mislocalized sites may shift the centered window and dilute enriched motifs.
  a. From the Progenesis phosphoproteomic output, extract peptide sequences containing differentially expressed phosphosites.
  b. Align identified differentially expressed peptide sequences by their phosphosite with ±7 peptide windows flanking each phosphosite.
    ***Note:*** Some identified phosphosites may not align perfectly during alignment for motif analysis. Verify placement against full peptide sequence.
  c. Submit aligned sequences in pLogo or iceLogo to generate peptide sequence motif logo.
    i. The logo plots *p*-value on the y-axis to show statistical significance of each residue at each position relative to the phosphosite-centered peptide window on the x-axis.
      ***Note:*** At the time of writing, the iceLogo web server is not operational, but the standalone software remains available for download (https://compomics.github.io/projects/iceLogo).
  d. Interpret the output logo data.
    i. Interpret the enriched phosphorylation motif directly or use additional tools to infer the candidate upstream kinases (e.g., PhosR, https://kinase- library.mit.edu/home, etc).
24. For general phosphoprotein functional enrichment analyses, follow steps 22a-c using the list of phosphoproteins.

**CRITICAL:** Pathway analysis of phosphoproteins strips phosphosite specificity. It reveals functional connection but does not indicate activation or inactivation states.

***Note:*** Proteomics tools evolve rapidly. Check for updated databases and software versions before starting analysis.

***Optional:*** Tools like Cytoscape and STRING can also be used to identify/map potential protein-protein interaction networks.

**CRITICAL:** After proteomic and/or phosphoproteomic analysis, it is crucial to **validate protein-level and functional findings** using orthogonal approaches such as western blots, immunohistochemistry, or another relevant assay. In the associated study,^1^ the proteomic/phosphoproteomic analyses implicated CaMK signaling and identified increased CaMK1α in the chronic-pain VTA. This finding was independently validated by immunohistochemistry, including increased CaMK1α within TH-positive VTA dopamine neurons. Acute CaMK1 inhibition with CS640 also reversed a subset of the pain-associated phosphorylation changes. These examples illustrate how outputs from the workflow can be used to generate hypotheses for subsequent experimental validation.

## Expected outcomes

Following in-gel digestion of 50 μg VTA lysate from pooled bilateral punches using the mass spectrometry setup described here, the workflow will typically identify up to 6,260 proteins per sample supported by at least two unique peptide sequences. When there are differences between experimental groups, the resulting dataset will support separation of experimental conditions in principle component analysis and hierarchical cluster heatmaps when biological variance exceeds technical variance. Hierarchical clustering heatmaps group samples and proteins by similarity, revealing distinct expression profiles across experimental groups and identifying proteins that similarly vary across conditions. These analyses determine the similarities and differences between the experimental groups, as well as the overall reproducibility of the experiment. Protein yield depends on digestion completeness, instrument performance and acquisition settings, and tissue complexity. Lower-than-average values usually indicate a technical limitation of that run rather than a biological difference between samples.

Phosphoproteomic analysis of 200–400 μg lysate following SMOAC enrichment generally yields, on average, 3,680 unique phosphopeptides, making up ∼75% of the total unique peptide sequences, that similarly support clustering of biological replicates and separation by experimental conditions when significant differences exist. As expected for eukaryotic phosphoproteomes, the phospho-amino-acid distribution is predominantly phosphoserine (pSer) followed by phosphothreonine (pThr) (Figure 4B).^19^^,^^20^ The detection of less abundant phosphorylation residues, such as phosphotyrosine (pTyr), depends on MS instrument sensitivity and may vary when using ultrasensitive mass spectrometers. Phosphopeptide yield depends on the same factors that affect protein yield as well as the mass of digest loaded onto SMOAC and on enrichment efficiency.

Motif enrichment analysis reveals overrepresented kinase consensus sequences, identifying overrepresented phosphorylation motifs that may implicate candidate upstream kinase families without requiring phospho-specific antibodies. For example, in the VTA dataset underlying this protocol, motif analysis identified enrichment of a proline-directed phosphorylation motif consistent with candidate MAP kinase signaling, while functional analyses implicated mitochondrial/metabolic, cytoskeletal, synaptic, glutamatergic, autophagic, and cellular-stress processes. SynGO restricts enrichment analysis to synapse-specific compartmentalization and biological processes, generating sunburst plots that visualize enrichment and protein counts for these synaptic localizations. The use of multiple downstream analyses should be treated as standard outputs of the workflow rather than optional post-processing to gain insight into larger patterns of coordinated signaling changes.

## Limitations

Pooled samples (2–3 bilateral VTA punches) are required to reach the protein input threshold for both global proteome and phosphoproteomic analysis. Tissue pooling increases the material available from small, anatomically constrained regions such as the VTA and reduces technical variability, enabling reproducible survey measurements. However, pooling necessarily sacrifices animal-level resolution: biologically meaningful inter-animal differences are averaged, biological heterogeneity cannot be assessed, and pooled measurements cannot support inference about individual animals. Pooling may also influence proteomic and phosphoproteomic readings by increasing concentrations of proteins and phosphopeptides of collected regions. This protocol is therefore hypothesis-generating for regional molecular states; findings should be validated in single-animal or orthogonal assays where animal-level inference is required. While current iterations of our pipeline require pooling, the emergence of ultrasensitive MS platforms suggests that single-animal VTA analysis may become feasible as technology evolves. In-gel fractionation enhances depth, but the inherent dynamic range of the proteins with low abundance (<100 copies/cell) may not be detected. In such cases, deep fractionation, targeted parallel reaction monitoring, or selected reaction monitoring approaches are recommended when looking for specific targets. Bulk tissue collection, as used in this pipeline, may contain tissue and cell types outside the intended regions. Laser capture microdissection (LCM) or fluorescence-activated cell sorting (FACS) could improve cell-type specificity, but each introduces significant practical limitations. LCM yields small amounts of sample, making it difficult to collect sufficient protein for PTM enrichment workflows, and the extended time required for sectioning and dissection would allow endogenous kinases and phosphatases to remain active, thereby altering the phosphoproteome before collection. FACS requires enzymatic tissue dissociation, which can activate stress signaling cascades and perturb phosphorylation states, meaning the sorted cells would no longer reflect the in vivo signaling state of the intact tissue. Additionally, since the 1 mm biopsy punch may include regions outside the VTA and due to the heterogeneity of the neuronal types in the VTA, the samples collected will represent the overall profile of all the collected tissue. This means that interpretation of these findings needs to be applied broadly to the collected tissue and the experiment must be labeled as exploratory or hypothesis-generating.

Data interpretation is subject to common proteomic challenges, such as inconsistent protein identification across samples and the “protein grouping” problem, where a single peptide sequence maps to multiple proteins and complicates differentiation. Enrichment tools can occasionally return overlapping terms or broad categories that obscure more biologically relevant “driver” terms. The protocol uses g:Profiler to help isolate functionally relevant terms. While user-friendly platforms such as g:Profiler, SynGO, and DAVID streamline analysis, specialized tools like PhosR and PTM-SEA have steeper learning curves and may require additional software depending on the use case. Enrichment analyses and kinase-motif predictions are hypothesis-generating approaches. They identify candidate pathways and upstream kinases for follow-up testing but do not by themselves demonstrate pathway activation. Orthogonal validation—e.g., immunoblotting/IHC of specific phosphosites, kinase perturbation, or targeted experiments—is required before mechanistic conclusions are drawn. Finally, since many of these tools are hosted online, they are susceptible to discontinuation when servers are no longer maintained, and it is critical to check availability before experimentation.

## Troubleshooting

### Problem 1

Low protein yield from VTA (related to Step 4).

### Potential solution

Low protein yield usually stems from incomplete homogenization or tissue loss during collection. Increase sonication time or pulse number to ensure full lysis and verify the biopsy punch fully penetrates the slice. Use of other homogenization methods, like bead-beating homogenizers, may provide further benefit for extracting more total protein simultaneously. If this is not sufficient, you can adjust the components of the lysis buffer to improve lysis or you can pool additional bilateral punches per sample. Alternative or additional lysis approaches, including chaotropic agents (e.g., urea) and alternative mechanical disruption (bead-beater, mortar and pestle, or rotor-stator homogenization) should also be considered. Changes to lysis composition should be validated for compatibility with the downstream MS workflow rather than applied routinely.

### Problem 2

Issues with Gel Separation and Trypsin Digestion (related to Steps 6–9).

### Potential solution

Poor gel separation or low trypsin digestion efficiency can lead to suboptimal peptide recovery and poor sequence coverage. Poor separation is frequently attributed to the use of aged SDS-PAGE reagents or incorrect voltage settings during electrophoresis. To resolve this, prepare fresh running buffer and verify the power supply settings. Regarding digestion, low efficiency is often the result of expired trypsin aliquots or incomplete destaining of the gel pieces, which inhibits enzyme activity. We suggest using fresh trypsin aliquots and ensuring the gel pieces are completely clear of stain before initiating digestion.

### Problem 3

Low Peptide Recovery and Enrichment Failure (related to Step 15).

### Potential solution

Low peptide recovery from ZipTips is typically caused by overloading or incomplete elution. Reduce the load volume and perform a double elution to ensure solvent fully contacts the resin. If phosphopeptides appear in the total proteome run but are absent after SMOAC, the enrichment failed. Verify that SMOAC reagents are fresh, check the binding-buffer pH, and minimize transfer steps to reduce sample loss.

### Problem 4

LC-MS/MS Technical Issues and Data Quality (related to Steps 11–13).

### Potential solution

High carryover, retention-time drift, and elevated missing values can severely compromise quantitative accuracy. To mitigate carryover, insert blank runs between high-concentration samples, and extend the high-organic wash step. To correct retention-time drift, verify column-oven temperature stability and ensure gradient consistency. To minimize missing values, standardize sample preparation protocols and dilute complex samples to reduce matrix-induced ion suppression. When working with Progenesis, chromatographic alignment is key to success, therefore, sample preparation must be consistent, and the column must be fresh.

### Problem 5

Low Phosphosite Identification and Localization (related to Steps 14–16).

### Potential solution

A low number of differentially phosphorylated sites or ambiguity in site localization can limit the biological conclusions of the study. Insufficient biological replicates (n < 4–6) or high variability in sample handling often lead to a lack of statistical significance. We recommend increasing the number of replicates per group. Additionally, low phosphopeptide recovery could be a sign that there are too many agents in the sample that are interfering with the SMOAC sample prep or that phosphatase activity is still present. For interference you may need to adjust the RIPA lysis buffer and lower the concentration of the detergents present. Make sure to check that your phosphatase inhibitors are not expired and are added immediately before use to ensure phosphopeptides remain phosphorylated. Of course, the more starting material, the better the depth of phosphoproteome coverage.

### Problem 6

Peptide sequences mapping to multiple proteins (related to Step 13).

### Potential solution

Identified peptide sequences overlap with more than one protein in the database and make it impossible to assign uniquely. The frequency of protein grouping varies among datasets because it depends on sequence homology, peptide coverage, database composition, and search parameters. Grouped proteins should be identified in the analysis output and interpreted cautiously at the individual-protein level. When interpreting results, note the proteins that are grouped together. Often these proteins group in a functionally related manner. Protein grouping should not affect pathway enrichment results but does affect individual protein-level interpretation.

### Problem 7

Few enrichment annotations for protein list (related to Step 22).

### Potential solution

If g:Profiler yields no significant terms at p < 0.05, a common outcome for small input lists (<20 genes), consider reducing the minimum term size to 3 proteins. If no terms meet the prespecified significance threshold, report that result and consider complementary annotation resources (e.g., DAVID) or larger input lists. Similarly, sparse SynGO annotations are expected when datasets contain substantial non-synaptic components (e.g., mitochondrial or cytosolic proteins), given that SynGO covers only ∼40% of the proteome; in such cases, complement SynGO with standard GO enrichment via g:Profiler to capture broader biological processes rather than relying solely on synaptic-specific curation.

### Problem 8

.raw import issues (related to Step 13).

### Potential solution

If.raw files fail to import or process correctly, first verify file size and acquisition completion. A common failure mode is copying from a drive before the transfer has completed, producing a truncated file that fails import; repeat the transfer if so. If a fatal error during acquisition stopped a run prematurely, re-run that portion of the sample—retain sufficient sample volume for re-analysis in case a run fails. Review instrument error logs, confirm compatibility between the acquisition file and analysis-software version, and inspect chromatographic alignment. Always retain the original.raw files before attempting repair, conversion, or reprocessing.

### Problem 9

Analysis-level data issues – batch effects, missing-value inflation, normalization artifacts, unexpected PCA clustering (Related to Steps 13, 18–21).

### Potential solution

These problems come from run order and experimental design rather than a failed wet-lab step, and they affect every downstream analysis built on the normalized feature matrix, from PCA and volcano plots to enrichment and motif analysis.

**Batch effects** are minimized by design. Stagger sample order across sets instead of running each condition together, use fresh solutions, and keep the LC/MS calibrated and clean (annual preventive maintenance). Where feasible, use multiple animal/sample batches as biological replication, and check PCA for batch structure before interpreting biology.

**Missing-value inflation** has several sources: low-abundance features near the detection limit, run-to-run retention-time shifts, and sample-specific problems such as a bad injection, a clogged column, or low loading. Alignment/co-detection quantitation (as in Progenesis) reduces but does not eliminate missing values, and this protocol filters low-quality features and does not impute them. First determine whether missingness, that is, the pattern or way data values are absent, is technical or biological, and whether it concentrates among low-abundance features (left-censoring). If a later analysis requires a complete matrix, an imputation method matched to the censoring type can be applied after filtering, as an optional analysis choice.

**Normalization** assumes that most features do not change between conditions and that the remaining run-to-run differences are a constant scaling factor. Artifacts appear when that assumption breaks. If a large fraction of features changes genuinely between groups, the algorithm reads the real biological shift as a technical offset and removes it, attenuating true fold-changes. Systematic differences in total loading mask true differences, and a few extreme outlier ions can over- or under-correct a whole run. Inspect the per-run normalization factors (values far from 1.0 deserve scrutiny) and compare PCA before and after to confirm that known biological structure is preserved. When a global biological shift is expected, normalize to a set of stable housekeeping proteins rather than to all peptide ions (a Progenesis option).

**Unexpected PCA clustering** usually signals cohort- or study-level structure, such as cohort/animal/batch differences, sample contamination, or inadequate cleaning between runs. Examine sample metadata against the PC loadings to find the driver before drawing biological conclusions.

### Problem 10

Bioinformatics servers are no longer maintained (related to Steps 22–24).

### Potential solution

Web-hosted tools are sometimes relocated or discontinued after publication, and an analysis step that depends on a specific server can become unusable. First, check whether a locally installable version exists. Many tools are distributed as standalone applications, R packages, or open-source code and keep working after their web servers are retired. Source archives may also persist on repositories such as GitHub or CRAN. If no maintained version is available, look for the tool’s associated publication that describes the analysis method. The publication may also be useful for identifying an alternative tool that performs the same or similar analysis. Papers citing the original tool are often the most direct route to a maintained substitute, and repositories such as bio.tools index equivalent software. Any substituted software should be verified as appropriate for the intended analysis and applied with statistical stringency equivalent to the original method, and the substitution and parameters should be documented. If no suitable alternative can be identified after these steps, the analysis itself may need to be reconsidered rather than forced through an unsupported substitution.

## Resource availability

### Lead contact

Further information and requests for resources and reagents should be directed to and will be fulfilled by the lead contact, Arthur C. Riegel (ariegel@arizona.edu).

### Technical contact

Technical questions on executing this protocol should be directed to and will be answered by the technical contact, Paul R. Langlais (langlais@arizona.edu).

### Materials availability

This study did not generate new unique reagents.

### Data and code availability

The datasets generated during this study are available at PRIDE: PXD072701 (https://www.ebi.ac.uk/pride/archive/projects/PXD072701).

## Acknowledgments

This work was funded by grants from the 10.13039/100000026National Institute on Drug Abuse through the University of Arizona Center of Excellence in Addiction Studies (CEAS; P30DA051355), with additional support from the Department of Pharmacology Comprehensive Center for Pain and Addiction (CCPA) in the College of Medicine at the 10.13039/100007899University of Arizona. 10.13039/100018491The University of Arizona Health Sciences Quantitative Proteomics Laboratory is acknowledged for MS-based proteomic and phosphoproteomic support. Graphical abstract illustrations were created using BioRender.com.

## Author contributions

C.C.D., writing/editing, tissue collection and processing, and data analysis; P.R.L., writing/editing, mass spectrometry experiments, and data analysis; T.W.V., editing; A.C.R., writing/editing, tissue collection, and data analysis.

## Declaration of interests

The authors declare no competing interests.
